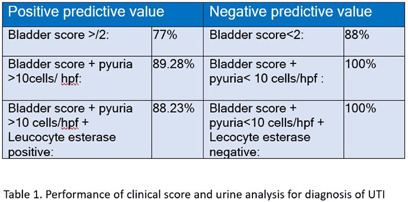# Optimizing Urine Culture Utilization in the Emergency Department, a Study from South India

**DOI:** 10.1017/ash.2025.308

**Published:** 2025-09-24

**Authors:** Krishna Suresh, Dheeraj Mohan, Rajalakshmi Ananthanarayanan, Vettakkara Kandy, Muhammed Niyas

**Affiliations:** 1 KIMSHEALTH

## Abstract

**Background:** Inappropriate urine culture can lead to unnecessary antibiotic use, antimicrobial resistance, increased healthcare costs, and resource strain. Ensuring the appropriate use of urine cultures aligns with principles of diagnostic stewardship. **Methods:** Urine cultures ordered from ED in our hospital, for patients who were admitted during July and August 2024 were retrieved from the electronic medical records. Symptoms score based on IDSA guideline (Figure 1) and BLADDER score (Figure 2) were correlated with urine analysis (URE) and cultures for appropriateness. **Results:** Among 267 urine culture orders that were reviewed, 61 patients were excluded due to indwelling catheter, high-risk neutropenia, recent urological procedures, pregnancy, or recent renal transplantation. The median age of study population (n=206) was 64 years. 50.50% were women. 97 (47.3%) had significant pyuria, and 105 (50.97%) had a positive leukocyte esterase (LE), nitrite positivity was low 13 (6.3%). LE had better correlation with pyuria and culture positivity when compared to urine nitrites. Only 46 patients (22.3%) had culture positivity. Imaging evidence supportive of urinary tract infection was noted in 18 patients. Among 206, only 102 cultures (50.48%) were appropriate as per IDSA guidelines. Inappropriate cultures were ordered for fever (59.6%) without localisation, abdominal discomfort (8.6%), urinary frequency (2.8%), haematuria (1.9%), incontinence (0.9%). 10% were sent as part of order sets, who were asymptomatic and had no significant pyuria or cultures positivity. Among 87 patients with a BLADDER score ≥2, 95.4% of cultures were appropriate, 64.3% had significant pyuria, 36.8% had culture positivity. Among 119 patients with a score < 2, 15.9% of cultures were appropriate, 34.5% had significant pyuria, 11.8% had culture positivity. Positive predictive value (PPV) of BLADDER score for UTI was 77.0%, 89.3% along with pyuria and 88.23 % when combined with pyuria and positive LE. Negative predictive value (NPV) of BLADDER score for UTI was 88.2%, 100% along with absence of pyuria and 100% when combined with absence of pyuria and negative LE (Table 1). Based on our study the proposed algorithm for ordering urine culture, after excluding the high risk group is depicted in the Figure 3. **Conclusion:** Our study showed 50% of urine culture as inappropriate. BLADDER score can be a useful bedside screening tool for deciding urine culture, PPV and NPV increase when combined with presence or absence of pyuria and LE. Implementing a diagnostic stewardship protocol in urine culture has the potential to improve culture appropriateness, reduce unnecessary antibiotic use.

**Figure:**
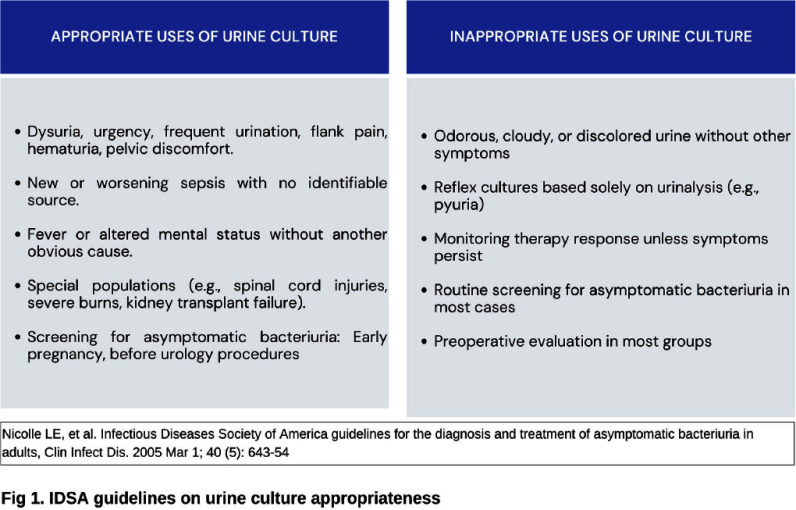

**Figure:**
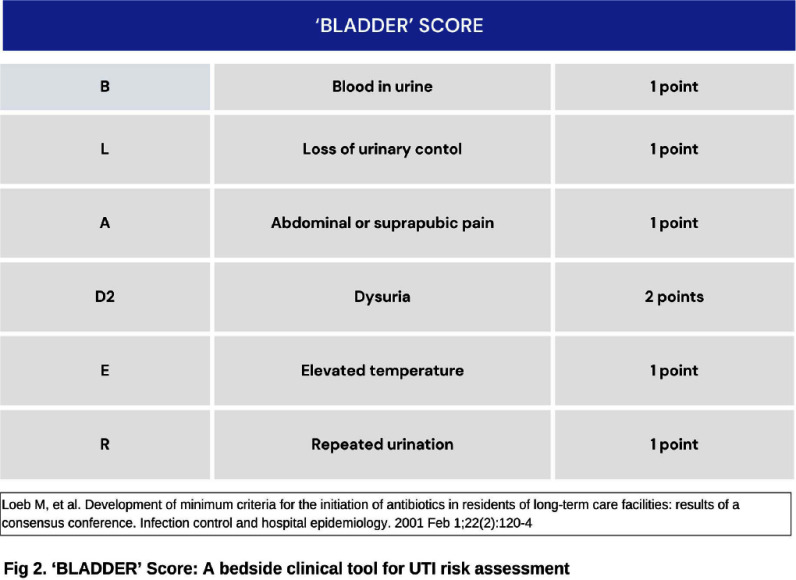

**Figure:**
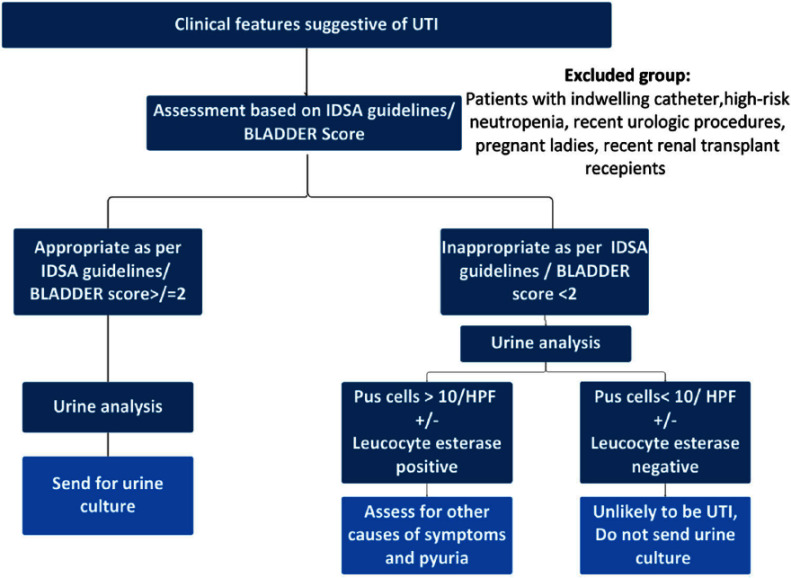

**Figure:**